# Survey of Impression Materials and Techniques in Fixed Partial Dentures among the Practitioners in India

**DOI:** 10.1155/2013/430214

**Published:** 2013-04-22

**Authors:** Arvind Moldi, Vimal Gala, Shivakumar Puranik, Smita Karan, Sumit Deshpande, Neelima Neela

**Affiliations:** ^1^Department of Prosthodontics, HKE's SN Institute of Dental Sciences and Research, Gulbarga 585105, Karnataka, India; ^2^Department of Periodontology, Adesh Institute of Dental Sciences, Bhatinda 151109, India; ^3^Department of Prosthodontics, Pandit Deendayal Upadhyay Dental College and Hospital, Solapur 413255, Maharashtra, India; ^4^Department of Periodontology, Haldia Institute of Dental Sciences and Research, Banbishnupur, Haldia 721645, West Bengal, India

## Abstract

*Objective*. Anecdotal evidence suggests that impression materials and techniques used in general dental practice for fixed partial dentures vary from those taught in dental schools. The aim of this survey was to integrate impression techniques evolved all over the years for fixed partial dentures and to know the techniques and materials which are used in the present day by the practitioners. *Materials and Methods*. A total of 1000 questionnaires were sent to various practitioners in India, out of which 807 questionnaires were filled. *Results*. The results showed that 84.8% of prosthodontists (65.56%, urban areas) use elastomeric impression materials as well as irreversible hydrocolloids and 15.2% use irreversible hydrocolloid only. Amongst other practitioners, 55.46% use irreversible hydrocolloid (45%, rural and semiurban areas) and 44.54% use elastomeric impression materials. Elastomeric impression technique practiced most commonly is putty reline with/without spacer (77.2%); other techniques are multiple-mix and monophase techniques. *Conclusion*. The ideal materials, technique, and armamentarium are required for the long-term success of the treatment for fixed partial denture. Also, if the ideal procedure is not followed, it will lead to a compromised fit of the final prosthesis and failure of the treatment.

## 1. Introduction

 Prosthodontics, as a speciality, has evolved abundantly in past few years. Materials and technological advances keep changing the face of every field every day. Twentieth century witnessed remarkable changes with regard to human longevity worldwide, and the twenty-first century is set to carry forward the gains in longevity further, both in the developing word and the developed world [1]. Various impression materials and techniques came into use since times earlier till today for fixed partial dentures, and all of them have some advantages and disadvantages and are suitable for specific conditions.

 This study used a questionnaire-based survey to assess and know the impression materials and techniques for fixed partial dentures that are being followed by the practitioners of India.

## 2. Materials and Methods

A confidential questionnaire was designed to assess the details of the impression materials and techniques in fixed partial denture. This questionnaire was initially sent to a group of 10 dentists, and a pilot study was carried out to check the contents and administrative aspects. Then the questionnaire was sent to 1000 dentists in India through e-mail, post, and handing it personally as well. All dentists were contacted regardless of age. An accompanying letter described the aims of the study and how the data would be used. Dentists were reminded by telephone 1 month after the initial mailing. Identification of individual respondents was not required to assure confidentiality.

## 3. Results

 A total of 1000 questionnaires were sent to various practitioners all over India out of which 807 questionnaires were filled. The results were categorized into metro places and nonmetro places. Metro places included were Mumbai, Bangalore, Chennai, Hyderabad, Jaipur, Delhi, Kolkata, and Ahmedabad. The data were collected anonymously, and so the results from the study could not be analyzed with regard to the year or place of graduation for individual dentists.

 Out of the 807 dentists who responded to the questionnaire, 33.33% were prosthodontists and 66.67% were nonprosthodontists. Participation from the metro areas was 58.21%, and it was 42.79% from the nonmetro areas. The survey results show the following.

**Figure 1:**
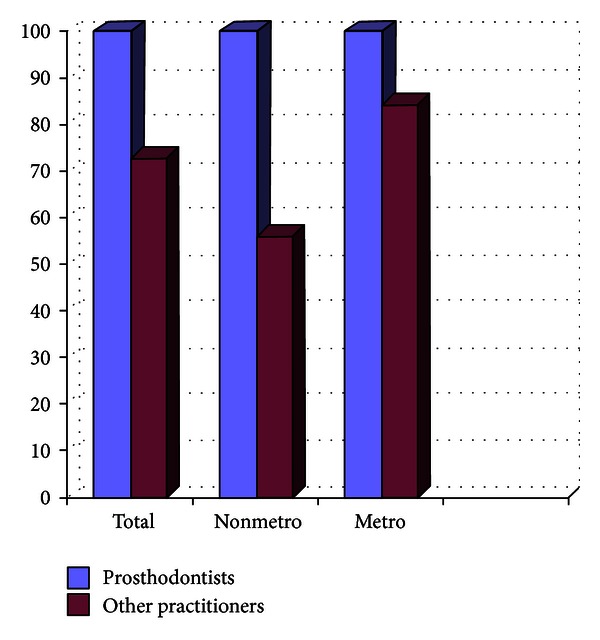
Graph showing the % of practitioners using alginate for diagnostic impressions.

**Figure 2:**
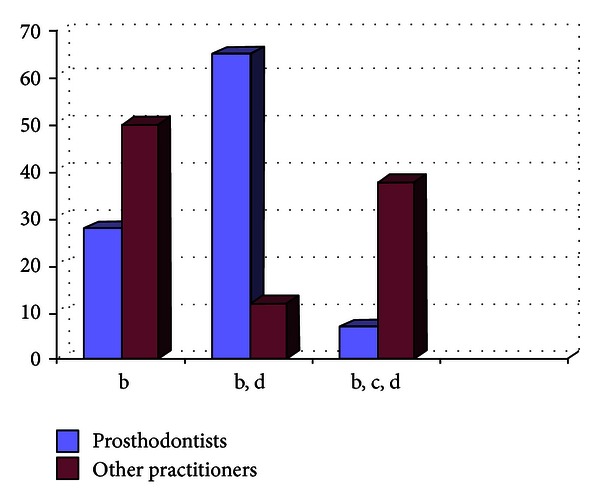
Graph showing the type of impression tray being used by the practitioners in %.

**Figure 3:**
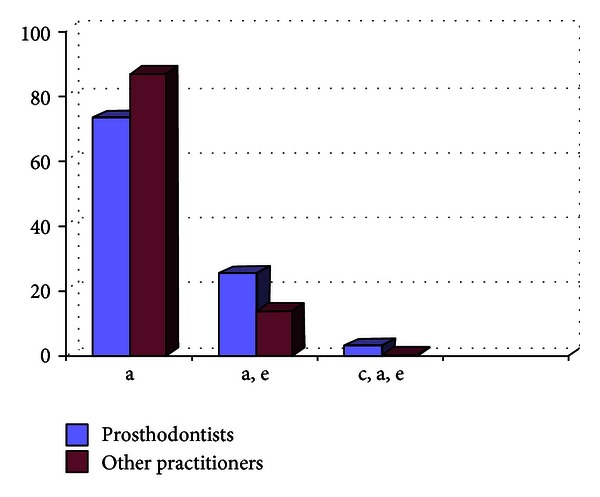
Graph showing the usage of gingival retraction materials by the practitioners (in %).

**Figure 4:**
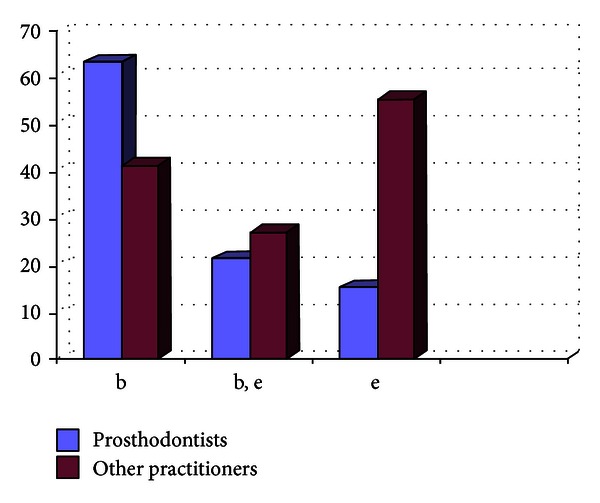
Graph showing the impression material being used by the practitioners (in %).

**Figure 5:**
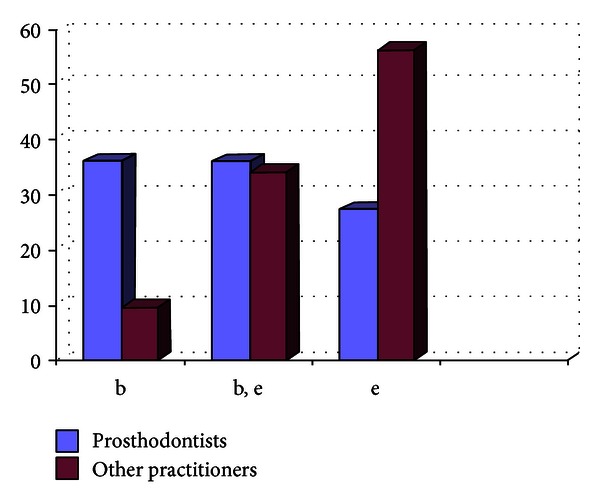
Graph showing the impression material being used by the practitioners (in %) in nonmetro areas.

**Figure 6:**
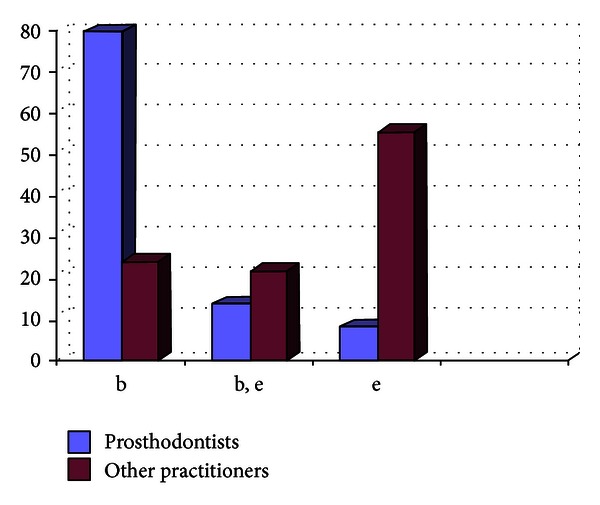
Graph showing the impression material being used by the practitioners (in %) in metro areas.

**Figure 7:**
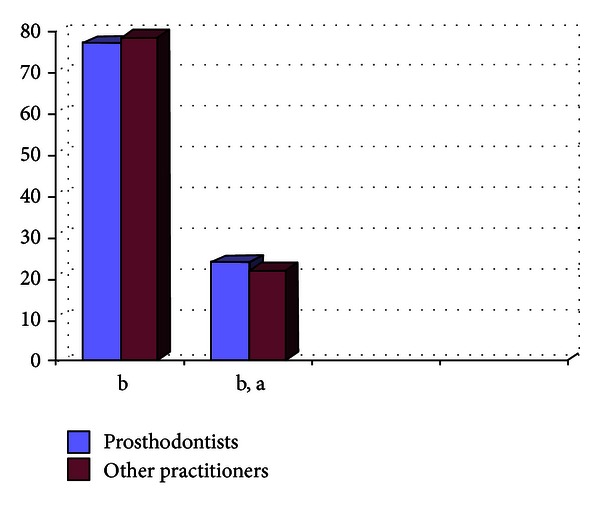
Graph showing the impression technique being followed by the practitioners (in %) for elastomeric impression materials.

**Figure 8:**
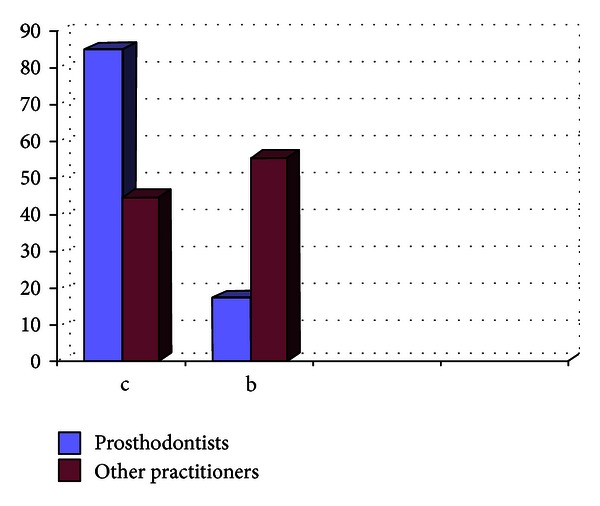
Graph showing the material used for pouring the impressions by the practitioners (in %).

1. There are 29% practitioners who do not take diagnostic impressions and proceed with the tooth preparation after the clinical intraoral examination, and majority of them are from the nonmetro areas (Figure 1).
2. Amongst the prosthodontists, 28% use only full arch impression trays, 62% use full arch and special trays, and 10% use full arch, partial arch, and special trays. Amongst the other practitioners, 49% use only full arch impression trays, 12% use full arch and special trays, and 39% use full arch, partial arch, and special trays (Figure 2).
3. 72.8% of practitioners use gingival retraction cord, 24.8% use gingival retraction cord, Expasyl, and gingifoam, and 2.4% use laser, gingival retraction cord, Expasyl, and gingifoam (Figure 3).
4. Amongst the prosthodontists 63.2% use addition silicone, 21.6% use addition silicone and alginate, and 15.2% use only alginate. Amongst other practitioners, 41.33% use addition silicone, 26.86% use addition silicone and alginate, and 55.43% use only alginate (Figure 4). In the nonmetro areas, amongst the prosthodontists, 36.17% use addition silicone, 36.17% use addition silicone and alginate, and 27.66% use only alginate, and amongst the other practitioners, 9.5% use addition silicone, 34.25% use addition silicone and alginate, and 56.16% use only alginate (Figure 5). In the metro areas, amongst the prosthodontists, 79.49% use addition silicone, 12.82% use addition silicone and alginate, and 7.7% use only alginate, and amongst the other practitioners, 23.53% use addition silicone, 21.57% use addition silicone and alginate, and 54.91% use only alginate (Figure 6).
5. Amongst the prosthodontists, 76.41% use putty reline technique with and without spacer and 23.58% use putty reline with/without spacer and single-mix technique. Amongst the other practitioners, 78.21% use putty reline technique with and without spacer and 21.79% use putty reline with/without spacer and single-mix technique (Figure 7).
6. Amongst the prosthodontists, 84.8% use type IV stone and 15.2% use type III stone. Amongst the other practitioners, 44.58% use type IV stone and 55.43% use type III stone (Figure 8).

## 4. Discussion

 The questionnaire results were assessed in general, and it was found that the recommended materials and techniques were followed by most of the prosthodontists but not by most of the general practitioners. Also, more recommended materials were used by the practitioners in the metro areas.

 Diagnostic impressions are of utmost importance for the treatment planning in fixed partial dentures. The diagnostic models when assessed will give the treatment outcome that is planned and any other treatment if required before proceeding with the fixed partial denture treatment, for example, enameloplasty of the opposing supraerupted tooth or uprighting of the abutment tooth, and so forth. 

 The impression trays used by many practitioners are the full arch impression trays. The full arch impression tray has many advantages as it records complete arch and the practitioner can make the impression with proper control over the setting time of the impression material unlike the dual arch impression tray. The dual arch impression tray is technique sensitive as the clinician needs to record both arches in the limited working time with the proper recording of the prepared teeth with the light body material. But the advantage is less time required for impression making as both arches are recorded simultaneously. Special trays are the best impression trays with the advantages of good confirmation to the arch, requirement of lesser material, and being economical as well. The partial arch tray is a poor choice for impression making as full arch recording is mandatory for proper mounting of the models, and further fabrication of the prosthesis depends on this mounting [2].

 Gingival retraction cord is being used since times earlier for gingival retraction to make impressions in FPD. The haemostatic agents are also used along with it to achieve desired hemostasis. The advantage of using a cord is that it is inexpensive and can achieve varying degrees of retraction. But, cords can be painful and uncomfortable for the patient. Also the sulcus collapses soon after the removal of the cord; that is, it might rupture the epithelial attachment. Hemostasis achieved is limited, and the placement of the cord in the sulcus takes time. An electrosurgery unit may be used for tissue removal before impression making. Electrosurgery is not recommended as the concentrated electrical current at the tip of electrodes can generate heat, which may cause osseous or mucosal necrosis, and also there is a potential for gingival recession after treatment [3, 4]. The consistency of Expasyl is especially formulated so as not to damage the healthy periodontium; the phenomena of gingival recession or bone resorption are thus avoided. Gingival retraction is obtained by a single application of Expasyl in the sulcus. On contact with crevicular fluid, this material provides mild displacement of the gingiva within two minutes [5, 6]. Expasyl, easily visible owing to its color, is simply eliminated by an air and water spray, and a dry and widely opened sulcus is then obtained. It is painless when used on a healthy periodontium. Absence of bleeding or oozing allows achieving a perfectly dry sulcus [7]. 

For impression making, elastomeric impression materials are the most superior in terms of recording finish lines and the surface detail of the prepared teeth; the disadvantages are delayed poring for addition silicone, difficulty in recording the arches with undercuts for polyether, and so forth [8–14]. Amongst the hydrocolloids, laminate technique, that is, the agar alginate technique, is better than using agar or alginate individually as agar will record the prepared teeth accurately and the remaining arch is recorded with alginate [15]. 

 The results clearly show that only alginate is being used by a large percentage in the nonmetro areas in spite of the proven fact that elastomeric impression materials are better than alginate for impression making, the reason being the cost which is higher for elastomeric impression materials. 

 For the technique of impression making, the single-phase (monophase) technique is faster and easier to use. The putty reline with spacer technique requires the use of spacer and is faster than using the putty reline without the spacer as in the latter; the space needs to be created for the light body syringe material using a putty cutter. 

 The impressions should be poured in type IV stone owing to its obvious higher mechanical properties as compared to type III stone. The final prosthesis accuracy of fit depends on this factor as well. 

## 5. Conclusion

 The ideal materials, technique, and armamentarium are required for the long-term success of the treatment for fixed partial denture. Single tooth when prepared and cemented with crown is at 3% risk for caries and endodontic failure and the abutment teeth prepared for multiple-unit FPD, are at 15% risk for caries and endodontic failure. Also, if the ideal procedure is not followed, it will lead to a compromised fit of the final prosthesis and failure of the treatment. 

## 6. Questionnaire for Impression Making in Fixed Partial Denture

(1) Which material do you routinely use for diagnostic impressions before tooth preparation?
  (a) Irreversible Hydrocolloid or alginate
  (b) Other (Please specify)
(2) Which tray are you using for making the impression after tooth preparation?
  (a) Dual arch tray (metal/plastic)
  (b) Complete arch (metal/plastic)
  (c) Sectional tray (metal/plastic)
  (d) Custom made acrylic tray
  (e) Other (Please specify)
(3) What do you practice routinely for gingival retraction?
  (a) Gingival retraction cord
  (b) Electrosurgery
  (c) Laser
  (d) Rotary curettage
  (e) Other (Please specify)
(4) If you use gingival retraction cord, is it used plain/with chemical and which chemical?
(5) Which material do you routinely use for impression after tooth preparation?
  (a) Condensation silicone
  (b) Addition silicone
  (c) Polyether
  (d) Polysulfide
  (e) Alginate Hydrocolloid
  (f) Agar Hydrocolloid
  (g) Agar-Alginate Combination
  (h) Other (Please specify)
(6) If you are using elastomeric impression materials, then which impression technique do you use?
  (a) Single mix (monophase) technique
  (b) Putty reline/Dual mix technique with spacer
  (c) Putty reline/dual mix technique without spacer
  (d) Multiple mix technique
(7) With what material is the cast poured?
  (a) Dental plaster (Type II)
  (b) Dental stone (TYPE III)
  (c) Dental stone High strength (TYPE IV)
  (d) Dental stone high strength, High expansion (TYPE V)
  (e) Other (Please specify)
   Name of the practitioner:
   BDS/MDS (if MDS please fill in the subject in which MDS is done):
   Year since practicing:
   Attached to which college (if any):

Note. Confidentiality of answers is assured as identification of individual respondent is not required, so please answer all questions as accurately as possible.
